# In Vitro Evaluation of Mechanical Fat Processing Devices: Impact on Adipocytes and Adipose‐Derived Stem Cells

**DOI:** 10.1155/sci/9150101

**Published:** 2026-06-30

**Authors:** Sheila Veronese, Riccardo Ossanna, Sara Ghazanfar Tehrani, Lorena Torroni, Patricija Kasilovska, Mario Goisis, Giamaica Conti, Domenico Amuso, Antonio Scarano, Andrea Sbarbati

**Affiliations:** ^1^ Department of Neuroscience, Biomedicine and Movement, Section of Anatomy and Histology, University of Verona, Verona, 37134, Italy, univr.it; ^2^ Saint Camillus International University of Health and Medical Sciences, Roma, 00131, Italy; ^3^ Luigi Vanvitelli University, Napoli, Italy; ^4^ De Clinic, Viale Regina Giovanna 39, Milan, 20129, Italy; ^5^ Department of Innovative Technology in Medicine and Dentistry, University of Chieti-Pescara, Via Dei Vestini 31, Chieti, 66100, Italy, unich.it

**Keywords:** ADSC, fat grafting, lipofilling, mechanical fat processing, mesenchymal stem cells, nonenzymatic fat processing

## Abstract

In the fat grafting procedure, the new European Regulations and the necessity to perform minimal manipulation processing require the verification of security and performance levels of each device and, in this particular context, functional and tissue responses. The aim of this study is to evaluate the integrity of adipocytes and adipose‐derived stromal/stem cells (ADSCs) after their processing using two new mechanical devices operating with a closed system. In this study, two new fat processing systems were assessed. We also considered the biological results of the processing and compared them with the results obtained from Coleman’s procedure, the golden standard procedure in fat processing. Histological evaluations revealed the preservation of the fat morphology after both processing types, with similar cellular yields of the extracted ADSCs. The adopted techniques enable the isolation of ADSCs with robust differentiation potential toward adipogenic, chondrogenic, and osteogenic lineages. Furthermore, adipose tissue samples exhibit high efficiency in extracellular vesicle secretion, indicating a promising potential for therapeutic and regenerative applications. The results suggest that the new systems allow the preservation of fat morphology, stemness, and regenerative potential. The novel processing technique proposed by the authors consists of a closed‐loop mechanical system that combines continuous filtration and emulsification of adipose tissue through saline washing, aimed at removing biological debris and residual pharmacological agents.

## 1. Introduction

Autologous fat grafting is widely used across regenerative, reconstructive, and esthetic medicine due to the ready availability of adipose tissue, its autologous compatibility, and the presence of mesenchymal stromal/stem cells (MSCs) enriched within adipose‐derived stromal/stem cells (ADSCs) that display multipotent differentiation potential.

The best medical device for any treatment is a device with which the surgeon feels most comfortable working. This means that a medical device may be perfect for one surgeon while another may prefer a different instrument. The fundamental thing is to obtain the best results for the patients, guaranteeing their highest level of satisfaction while always respecting adequate security levels. Accordingly, and to ensure results and safety, the European Union (EU) Medical Device Regulation 2017/745 and the In Vitro Diagnostic Device Regulation (EU) 2017/746, applicable since May 2021, introduced the obligation for a device to respect defined security levels and guarantee defined performance levels [1, 2]. These new norms have paramount repercussions on surgeons’ activities and the biomedical devices market.

In the present study, the authors considered the integrity of the results since to be commercialized, a device must pass security controls (CTRLs). In fact, it is difficult to verify the utility of a device. To achieve this goal, it is necessary to evaluate and, often, quantify the functional effect of the treatment performed with the device. However, it may also be required to consider the tissues’ response to the treatment. Thus, fat grafting may be included among the treatment types requiring both functional and tissue response evaluations.

Fat grafting represents a common approach in regenerative, reconstructive, and esthetic medicine [3]. The technique involves harvesting adipose tissue from a donor site and transferring it to another anatomical region. This tissue is preferred because it is readily available, suitable for autologous use, and contains MSCs and related subpopulations [4], which are abundant within ADSCs [5, 6]. These MSCs exhibit multipotent differentiation capacity [7]. However, prior to reinjection, processing is required since the ADSC concentration in raw lipoaspirate is lower than in native tissue. Processing aims to enrich ADSCs and facilitate reinjection [8, 9]. Historically, the most widespread approach remains Coleman’s centrifugation‑based protocol, which standardizes harvesting and preparation steps and is frequently considered a clinical “gold standard.” However, excessive centrifugal forces can rupture adipocytes, alter ADSC yield/viability, and introduce variability because reporting rpm without rotor geometry impedes reproducibility in terms of relative centrifugal force (RCF, ×*g*). In response, a variety of nonenzymatic mechanical systems (e.g., filtration, washing, emulsification, sedimentation, and shear‐based processing) have been developed to minimize damage and preserve cell functionality while facilitating reinjection; yet, protocols and performance claims remain heterogeneous across devices and studies.

Before 2017, ADSC isolation typically relied on enzymatic digestion using collagenase [10]. Regulatory changes introduced by the U.S. FDA in 2017 restricted collagenase use, classifying it as more than minimal manipulation [11]. Consequently, alternative mechanical processing techniques have emerged [12, 13]. Despite these innovations, Coleman’s centrifugation‐based protocol remains widely adopted [14]. Excessive centrifugal force, however, risks adipocyte rupture and reduced ADSC viability [15]. This concern has driven the development of mechanical, nonenzymatic devices for fat processing [16, 17].

Despite the numerous articles concerning these devices, the different clinical applications of fat grafting, and their results, there is a limited amount of data relating to the biological effects of fat processing. Moreover, these data are heterogeneous. Some articles compared different fat processing techniques [18], others fat processing devices [3, 19], others both methods and devices [20], collagenases [21], and centrifugation [22]. It is not yet clear which procedure is the best, whether a combination of methods may be the best, or whether one device may offer some advantages over the others for processing.

This study presents two new devices that perform different types of fat processing in a completely closed system. The biological preparation obtained after the processing was analyzed from a histological point of view to verify the devices’ functionality and biological effects. Despite the extensive literature on the clinical applications of fat grafting and the introduction of new devices, consistent biological evidence on how different mechanical processing methods affect adipocyte morphology, ADSC yield, and phenotype, as well as their proliferation, differentiation, and extracellular vesicle secretion, is still lacking. Existing studies vary widely in the techniques used, applied parameters, and reporting methods, making it difficult to compare results [23].

To address this gap, the present study evaluates two closed mechanical processing systems, Beauty and Lipo‐Stem Duo, based on continuous washing and progressive filtration, directly comparing them with the Coleman technique.

The aim of this study is to evaluate the integrity of adipocytes and ADSCs after their processing using two new mechanical devices operating with a closed system.

## 2. Materials and Methods

In 2023, 17 patients underwent facial and neck lipofilling surgery at the De Clinic in Milan, Italy. Seventeen female patients (23–56 years old) underwent face and neck lipofilling in 2023. Five procedures used Coleman’s method, and 12 used the Beauty‐Stem Duo or Lipo‐Stem Duo. Patients had no prior abdominal surgery at the harvesting site and no relevant metabolic, neoplastic, or pharmacological conditions.

**Figure 1:**
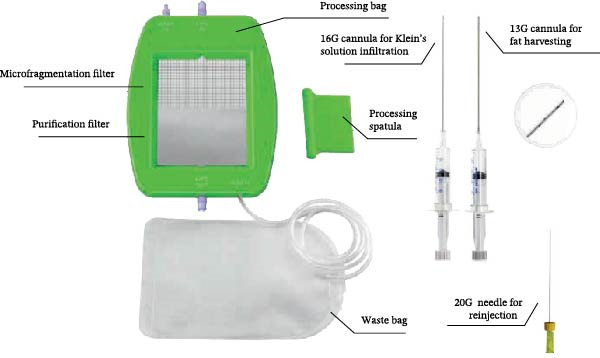
Beauty and Lipo‐Stem Duo processing systems.

Fat harvesting and processing were performed with Coleman’s procedure with five patients and the Beauty and Lipo‐Stem Duo processing systems (BIOPSYBELL S.R.L., Mirandola‐Modena, Italy) with 12 patients. Beauty‐Stem Duo (Identification Code: BETKIT‐005; CND: A019099) and Lipo‐Stem Duo (Identification Code: LPKIT‐002; CND: A019099) are equivalent systems used, respectively, in the esthetic and orthopedic fields. They include a 16G cannula for Klein’s tumescence solution infiltration, a 13G cannula for fat harvesting, a processing kit, and a 20G needle for reinjection (Figure 1).

The reprocessed fat was subsequently used for face and neck lipofilling. Morphological and functional characterization of the leftover processed fat was executed at the University of Verona. The single passages are detailed in the following subsections.

All the procedures were conducted in full compliance with the ethical norms and standards of the Helsinki Declaration of 1975, as revised in 1983. An informed written consent statement for data use and publication was obtained from all subjects.

### 2.1. Patients

The patients were all females aged 23–56 years old. They had not undergone previous surgery at the harvesting site that could have involved fibrosis or the presence of atrophic tissue. None of the patients were oncological subjects, obese, smokers, or had metabolic disorders. At the time of surgery, they were not assuming drugs.

### 2.2. Fat Harvesting

For Coleman’s procedure, an 8G cannula was utilized for fat harvesting, while a 13G cannula was preferred for the new processing systems. In both cases, harvesting was performed in the abdominal area.

A Klein’s solution was infiltrated in the harvesting area to induce local anesthesia and temporary vasoconstriction. This is necessary during harvesting to reduce bleeding, permitting tumescence generation. Klein’s solution was prepared by adding 0.5 mL of adrenaline 1 mg/mL and 20 mL of lidocaine 2% to a 250 mL saline solution.

To harvest 60 mL of lipoaspirate, 150–200 mL of Klein’s solution was needed (if a lipoaspirate greater than 60 mL was required, a larger volume of solution was prepared, with a ratio maintained between the infiltration and lipoaspiration volumes equal to 3:1).

2 mL of lidocaine 2% was infiltrated in the harvesting area. Then, a small incision was made to favor the entrance of the cannules. 60 mL of Klein’s solution was administered into the incision using a cannula of 16 G. Fat harvesting was executed after the analgesic solution had taken effect (7–8 min).

### 2.3. Fat Processing

Coleman’s standard procedure involves inserting the harvested fat into 10‐cc syringes. These are then centrifuged for 3 min at 3400 rounds per minute, generating a RCF of 1290.24 g. Subsequently, the top and bottom layers are removed from the syringes before the reinjection.

The Beauty and Lipo‐Stem Duo processing kits include two bags, one for actual processing and one for gathering waste products. The processing bag was divided into two parts. The upper part contains a mesh membrane >1000 µm that has a microfragmentation function. The lower part includes a 51 µm mesh membrane that has a cleaning function. The processing bag was filled with saline solution. Then, the lipoaspirate was inserted into the bag gently due to the presence of this saline solution. Excess solution is removed from the bag through a valve connected to the waste bag and located in the inferior part of the system. A continuous washing with saline solution is performed through a valve in the superior part of the system. When all the necessary lipoaspirate is inserted in the bag, a spatula is passed over the upper membrane to favor complete washing of the adipose tissue and elimination of blood, and damaged cells, intracellular oil droplets, cellular debris, that is to say, the biological waste products (BWPs), and also of the lidocaine used for infiltration. The second membrane retains the microfragmented lipoaspirate, which favors the elimination of BWPs produced during processing. When the lipoaspirate becomes yellow, the processing is completed, and the processed fat is ready to be reinjected.

Both procedures were applied. Face and neck lifting were performed. The nonnecessary fat (the leftover material) was collected for analysis. On the same day of harvesting, the sterilized plastic canisters containing fat were sent to the University of Verona. The canisters were shipped on dry ice to guarantee the samples’ preservation.

### 2.4. Histological Analysis

All the adipose tissue samples were fixed with paraformaldehyde 4% (Boster Biological Technology Co., Ltd.). After 20 min, the cells were washed with PBS 1×. When dried, they were embedded with an optimal cutting temperature (OCT) compound. Subsequently, samples were cryo‐sectioned into transversal slices of 14 µm thickness. By using the hood flow for 30 min, slices were dried and then kept at −20°C. To evaluate the morphology of the Coleman and Beauty and Lipo‐Stem Duo products, slides were rehydrated with distilled water for 2 min. Then, they were stained with Hematoxylin (Sigma–Aldrich, Milan, Italy) for 40 s. They were immediately gently washed with running water, stained again with 1/10 eosin (Sigma–Aldrich, Milan, Italy) for 10 s, and rewashed with distilled water. They were then dehydrated with alcohol at increasing concentrations (5 min at 80%, 5 min at 95%, and 5 min two times at 100%) and with xylene (applied twice for 5 min). Finally, a drop of Entellan (mounting medium) was added, and a cover slice was applied. An Olympus BX‐51 microscope (Olympus, Tokyo, Japan) equipped with a digital camera (DKY‐F58 CCD JVC, Yokohama, Japan) was used for analysis.

### 2.5. Enzymatic Digestion of Coleman and Beauty and Lipo‐Stem Duo Products for ADSCs Extraction

About 10 mL of lipoaspirates derived from Coleman’s technique and 10 mL obtained by the Beauty and Lipo‐Stem Duo processing systems were incubated with type I collagenase at 1 mg/mL concentration (GIBCO Life Technology, Monza, Italy). The products were then dissolved for 45 min at 37°C in Hank’s Balanced Salt Solution (HBSS, GIBCO Life Technology, Monza, Italy) with 2% of bovine serum albumin (BSA, GIBCO Life Technology, Monza, Italy). To neutralize the enzyme action, a Complete culture medium (Dulbecco’s Modified Eagle’s Medium [DMEM], Sigma–Aldrich, Italy), supplemented with 10% of fetal bovine serum (FBS), 1% of 1:1 penicillin/streptomycin (P/S solution), and 0.6% of amphotericin B (FBS, P/S solution, and amphotericin B were all from GIBCO Life Technologies, USA) was added. After this passage, the two samples underwent centrifugation at 3000 rpm for 5 min. The pellets of cells were incubated for 10 min at room temperature with 1 mL of erythrocyte lysis buffer 1× (Macs Miltenyi Biotec, Milan, Italy). The obtained cell suspensions were centrifuged again. Then, they were resuspended with 1 mL of a complete culture medium. Finally, a 70 µm nylon mesh was used to filter the cells. The resulting cells were seeded into a T25 flask.

### 2.6. Cellular Yield

The cells were extracted to quantify the cellular yield at 7, 14, and 21 days. The number of extracted cells was calculated by dividing the number of extracted free cells by the fat‐processed volume in order to evaluate the cellular yield. The number of living cells was calculated using the Trypan Blue exclusion assay in a CytoSMART counter (Automated Image‐Based Cell Counter, version 1.5.0.16380, CytoSMART Technologies B.V., Eindhoven, the Netherlands).

The extracted cells were seeded into a 25 cm^2^ T‐flask with a complete culture medium and incubated in a humidified atmosphere at 37°C with 5% CO_2_. The first medium change was performed 72 h after enzymatic digestion. Subsequently, the medium was changed every 48 h. Cellular growth was calculated with normalization by dividing the number of cells in the whole T‐flask after 7, 14, and 21 days of culture by the corresponding cellular yields. According to Dominici et al. [24], plastic adherence was evaluated at every passage to ensure the first criterion of MSCs definition.

### 2.7. Immunophenotype Analysis With Fluorescence‐Activated Cell Sorting (FACS)

According to Dominici et al. [24], specific surface antigen (Ag) expression was evaluated on cultured cells (cellular passage 4) obtained from standard enzymatic cell extraction of adipose tissue treated with Coleman’s protocol and with Beauty and Lipo‐Stem Duo. Triplicate samples were characterized by flow cytometry. The different cells’ products were centrifuged at 400 ×*g* (3000 rpm) for 6 min to perform cytofluorimetric analysis. The cell pellet was obtained. Then, it was resuspended in a complete culture medium and incubated for 10 min at room temperature with 1 mL of erythrocyte lysis buffer 1× (Macs Miltenyi Biotec, Milan, Italy). Thus, the sample was filtered through a 70 µm cell strainer. Subsequently, cells were washed with 1 mL of PBS and incubated (1 × 10^5^ for each tube) with conjugated antibodies on ice for 30 min. After incubation, centrifugation at 5000 rpm for 7 min was performed, and then the pellets were resuspended in 100 µL of PBS. The minimal criteria for identifying mesenchymal stem cells were considered when selecting the antibodies. In accordance with the recent joint statement of the International Federation for Adipose Therapeutics (IFATS) and Science and the International Society for Cellular Therapy (ISCT) [25], the antibodies tested were Comp‐PerCP‐Cyt5‐5‐A conjugated CD105 (dilution 1:5), Comp‐BV421‐A conjugated CD73 (dilution 1:5), Comp‐APC‐A conjugated CD90 (dilution 1:20), Comp‐BV650‐A conjugated CD45 (dilution 1:20), Comp‐PE‐A conjugated CD34 (dilution 1:5), Comp‐FITC‐A conjugated CD29 (dilution 1:5), and Comp‐BV785‐A CD44 (dilution 1:20). All these antibodies were purchased from BD Biosciences, Becton Dickinson Italy S.p.A., Milan. Immunophenotyping was performed through a Chant II FACS (BD, Becton Dickinson).

### 2.8. Differentiation Potential

The differentiation potential was evaluated in vitro for both the biological samples treated with the Beauty and Lipo‐Stem Duo processing systems and those treated with Coleman’s procedure. The latter were used as CTRLs. The cultured cells at passage 2 of expansion were employed for differentiation. To verify the third criterion of MSCs definition [24], three different types of differentiation were analyzed: adipogenic, chondrogenic, and osteogenic.

#### 2.8.1. Adipogenic Differentiation—Methods

To grow adherent cells, 5000 cells were seeded on a 12‐well plate containing one glass slide per well. The cells were incubated at 37°C, 5% CO_2_ for 24 h. Subsequently, the culture medium was replaced entirely by adipogenic media Basal Medium with SupplementMix of the Mesenchymal Stem Cell Adipogenic Differentiation Medium 2 kit (Sigma–Aldrich, Milan, Italy). To evaluate the adipogenic differentiation capacity, after 7 and 14 days of incubation, the cells were fixed with Baker’s fixative (Bio‐Optica, Milan, Italy) for 10 min at 4°C, washed with tap water for 10 min, and stained with Oil‐Red‐Oil solution (Bio‐Optica, Milan, Italy) for 10 min and Mayer’s hematoxylin (Bio‐Optica, Milan, Italy) for 5 min. Finally, the glass coverslips were mounted with Mount Quick Aqueous (Bio‐Optica, Milan, Italy).

#### 2.8.2. Chondrogenic Differentiation—Methods

About 1 × 10^6^ cells resuspended in 5 µl of complete culture media were seeded in a 12‐well plate. After 2 h, the chondrogenic media StemPro Osteocyte/Chondrocyte Differentiation Basal Medium with the StemPro Chondrogenesis Supplement of the StemPro Chondrogenesis Differentiation (GIBCO Life Technology, Monza, Italy) was added. Afterwards, the medium was changed every 3 days. After 1 and 14 days of incubation, cells were fixed with 4% formaldehyde (Bioptica, Milan, Italy) in phosphate‐buffered saline (PBS 0.05 M) for 30 min at 4°C, washed twice with distilled water, and stained with Alcian Blue solution (Merck KGaA, Darmstadt, Germany) for 40 min and with Nuclear Fast Red (Bioptica, Milan, Italy) for 20 min. Finally, after brief dehydration, the glass coverslips were mounted with Entellan (Merck KGaA, Darmstadt, Germany).

#### 2.8.3. Osteogenic Differentiation—Methods

An amount of 5000 cells was seeded on a 12‐well plate. A complete culture medium was added. The medium was substituted by the osteogenic media StemPro Osteocyte/Chondrocyte Differentiation Basal Medium with StemPro Osteogenesis Supplement of the StemPro Osteogenesis Differentiation Kit (GIBCO Life Technology, Monza, Italy) after 24 h. The osteogenic differentiation capacity was evaluated after 7 and 14 days of incubation. The cells were fixed in PBS 0.05M for 30 min at 4°C with 4% formaldehyde (Bioptica, Milan, Italy). Then, they were washed with distilled water twice. Subsequently, they were incubated for 2–3 min with Alizarin Red Solution (Merck KGaA, Darmstadt, Germany) and for 30 s with Mayer’s hematoxylin (Bio‐Optica, Milan, Italy). Finally, after brief dehydration, the glass coverslips were mounted with Entellan (Merck KGaA, Darmstadt, Germany).

#### 2.8.4. Image Acquisition

The bright‐field optical microscope Olympus BX‐51 (Olympus, Tokyo, Japan) and the digital camera DKY‐F58 CCD JVC (Yokohama, Japan) were used to observe and capture images of the stained cells. First, the slides were cleaned with ethanol, and then, they were placed on the slide holder of the microscope. Fifteen images were acquired for each slide using a 20× objective for quantifying lipid droplets and a 10× objective for quantifying calcified and collagen matrices. To standardize quantification, the acquired images contained 8 to 12 cells.

#### 2.8.5. Analysis

The custom‐designed ImageJ software plug‐in (National Institute of Mental Health, Bethesda, Maryland, USA) was used for semiquantitative analysis. For both the procedures (Coleman and Beauty and Lipo‐Stem Duo), 3 samples were considered. The differentiation was conducted in triplicate, and 15 images were evaluated for each replica. Following a systematic randomized protocol, a 10× objective was used. The entire area of the objective was considered a region of interest (ROI). In these areas, on the cells’ cytoplasm, the number of red spots (which coincide with lipid droplets) was counted to evaluate adipogenic differentiation, and the blue spots (which coincide with the chondrogenic depots) were related to chondrogenic differentiation. Red spots coincide with lipid droplets and blue spots with chondrogenic depots. The mean area of the red spots was calculated for adipogenic differentiation evaluation, the mean area of collagen aggregates was measured to evaluate chondrogenic differentiation, and the mean area of calcification deposits was considered to estimate osteogenic differentiation. Lipid droplets, chondrogenic clusters, and calcium deposits were assessed at 7 and 14 days.

### 2.9. Extracellular Vesicles (EVs)

The EVs’ production from cell samples was evaluated from the supernatant obtained after 11 days of ADSC culture. Briefly, MSCs were grown in a complete culture medium (DMEM, Sigma–Aldrich, Italy), supplemented with 10% of FBS (GIBCO Life Technologies, USA), 1% of 1:1 penicillin/streptomycin (P/S solution, GIBCO Life Technologies, USA), and, after 9 days, FBS was removed. Centrifugation at 3500 rpm for 7 min was performed. Then, a 0.22 µm nylon mesh was used to separate the supernatant parts. This permits the removal of cell debris. In accordance with the manufacturer’s instructions, the PureExo Exosome Isolation Kit (101Bio, Mountain View, CA, USA) was utilized to extract EVs.

The Nanoparticles Tracking Analysis System (Nanosight NS300) measured the EVs’ size and concentration. As a volume of 800 µl was required to perform the measurements, all samples were diluted with an appropriate amount of PBS. To obtain each measure, three 1‐min videos were captured. The acquisition conditions were a cell temperature of 25°C, a syringe speed of 20 µl/s, and a laser wavelength of 488 nm. These parameters were established by the manufacturer’s software (NanoSight NS300). Then, the Nanoparticles Tracking Analysis 3.4 software (Malvern Panalytic) was used to acquire the sample videos and perform their analysis.

### 2.10. Statistical Analyses

The normality of data distribution was assessed for each experimental group and time point using the Shapiro–Wilk test. The EV characterization was reported as mean ± standard error of the mean (SEM) to highlight the mean’s precision.

The median and interquartile range (IQR) were used to describe the central tendency and dispersion of the data, respectively.

Comparisons between the two treatments at each time point were performed using the Kruskal–Wallis test for independent samples and Dunn’s post hoc test for pairwise comparisons. A Bonferroni correction was applied by adjusting the significance threshold according to the number of comparisons to address the risk of Type I error arising from multiple tests.

The statistical significance level was set at *p*  < 0.05 for the overall test. STATA software, version 18 (StataCorp, College Station, TX, USA), was used for the analysis, while GraphPad Prism 7.03 for Windows (GraphPad Software, La Jolla, CA, USA) was used for data visualization and graph preparation.

## 3. Results

### 3.1. Histological Analysis and ADSCs Culture

**Figure 2:**
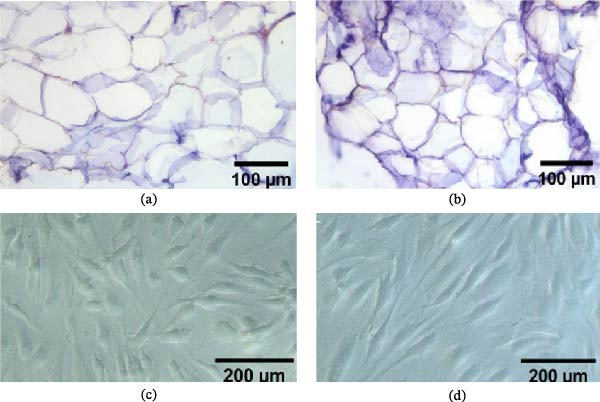
Histology of the fat processed with the two methods. (a) Fat obtained after Coleman’s processing; (b) Fat obtained after Beauty and Lipo‐Stem Duo processing. Both specimens present adipocytes with regular shape and diameter. The cytoplasmic membrane appears perfectly preserved. (Beauty and Lipo‐Stem Duo systems, *N* = 3; Coleman’s processing, *N* = 3). Culture of the ADSCs extracted from the fat tissue after fat processing using the two methods. (c) ADSCs extracted after Coleman’s processing; (d) ADSCs extracted after Beauty and Lipo‐Stem Duo processing. Both the figures refer to 7 days of cell culture at the P0 stage. In both cultures, ADSCs are spindle‐shaped, with normal morphology, without signs of stress, and in an active phase of proliferation. Cells appear perfectly attached to the culture plate (Beauty and Lipo‐Stem Duo systems, *N* = 9; Coleman’s processing, *N* = 8).

Considering that Coleman’s technique and Beauty and Lipo‐Stem Duo processing systems are different procedures, they can produce adipose tissue microfragments of various sizes. As shown in Figure 2, while the fragments obtained with Coleman’s procedure resulted in about 1000 µm in size, Beauty and Lipo‐Stem Duo generated fragments of smaller sizes. Despite this difference, in both cases, histological analysis highlighted a well‐defined and intact morphology of adipocytes in the fragments.

**Figure 3:**
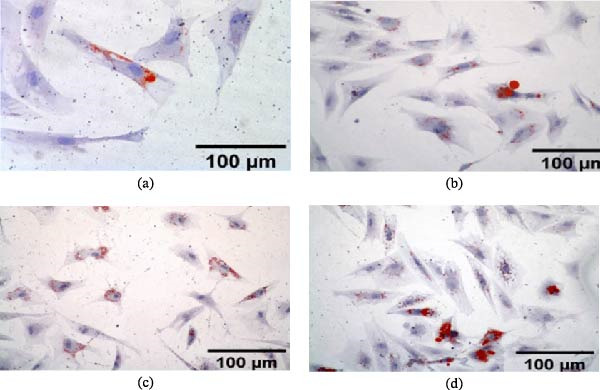
Immune phenotyping of adipose tissue treated with Beauty and Lipo‐Stem Duo systems. In panel (a), the figure shows the mesenchymal stem cell population’s characterization monitored immediately after the processing of lipoaspirate with Beauty and Lipo‐Stem Duo systems at passage 0 (P0). In contrast, in panel (b), the same characterization has been performed at passage 4 (P4) of the culture of the cell population isolated with the systems. Immune phenotyping of adipose tissue treated with Coleman’s protocol. Panel (c) shows the characterization of mesenchymal stem cells isolated from lipoaspirate processed following Coleman’s protocol at passage 0 (P0), while panel (d) shows the characterization of cultured stem cells at passage 4 (P4), isolated from lipoaspirates processed with Coleman’s protocol.

For both the processing methods, ADSCs appeared morphologically normal, vital, and perfectly plastic‐adherent to the culture plate (Figure 3).

### 3.2. Immunophenotype Analysis

Cultured ADSCs from immunophenotyping analysis revealed positivity for mesenchymal stem cell‐specific markers CD105, CD90, CD73, CD44, and CD29. The hematopoietic surface markers CD45 and CD34 resulted in negative results.

**Figure 4:**
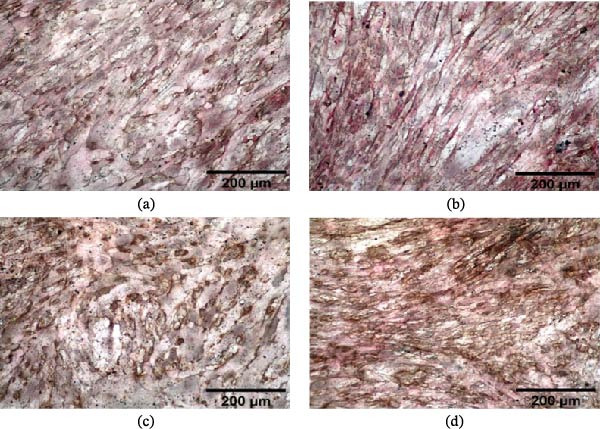
Histology of ADSCs cultured for adipogenic differentiation. (a) CTRL sample after 7 days of incubation; (b) sample treated with Beauty and Lipo‐Stem DuoTM after 7 days of incubation. Lipid droplets are evident in the cell cytoplasm in both cases. In sample b, they appear more numerous and broader than the droplets in sample a; (c) CTRL sample after 14 days of incubation; (d) sample treated with Beauty and Lipo‐Stem DuoTM after 14 days of incubation. Observations after 14 days of incubation confirm the results at 7 days of incubation. Lipid droplets in the subpanel (d) sample are more numerous and broader than in sample c (Beauty and Lipo‐Stem DuoTM systems, *N* = 3; Coleman’s processing, *N* = 3).

Figure 4 shows the results of the flow cytometry analysis to identify the presence of mesenchymal stem cells in lipoaspirates processed using the two different methods. It is possible to observe that, after both purification procedures, the lipoaspirates were characterized by mesenchymal stem cells expressing the typical markers with no substantial differences.

### 3.3. Cellular Yield and Growth Capacity

The cellular yield is related to the number of extracted cells after the two processes. Cellular growth permits these cells’ proliferative and growth capacity to be deduced. The analysis of the cellular yield dimensions revealed no significant differences between Coleman’s procedure and the Beauty and Lipo‐Stem Duo systems (*p* = 0.442). A statistical difference was noted comparing the cellular growth of the cells extracted through the two methods. A comparison of the two procedures at the 7‐ and 14‐day time points showed a statistically significant difference (*p* = 0.006). However, at the 21‐day time point, while there was a marginally significant difference between the procedures (*p* = 0.0415), this did not reach the threshold of statistical significance after correction for multiple comparisons according to Bonferroni; therefore, no superiority can be claimed at this time point. The DSCs obtained after processing with the Beauty and Lipo‐Stem Duo systems showed significantly higher growth capacity at days 7 and 14 compared to ADSCs extracted after Coleman’s procedure. At day 21, although a difference was still present, it did not reach statistical significance after Bonferroni correction.

### 3.4. Multilineage Differentiation

In Figure 4, it is possible to appreciate how all the samples showed adipogenic differentiation capability. This capability was different at different times. In fact, after 7 days of incubation, the cells treated with Beauty and Lipo‐Stem Duo had a higher mean amount of lipid droplets (147,2 ± 20,9) than the CTRL cells (88,5 ± 8,0), with a *p* ≤ 0.05;. In contrast, after 14 days of incubation, the CTRL cells presented a higher mean amount of lipid droplets (330 ± 31,1) with smaller areas (5,6 µm^2^ ± 0,6 µm^2^) concerning the droplet number of the cells processed with Beauty and Lipo‐Stem Duo (179,4 ± 19,9), and area (10,4 µm^2^ ± 0,78 µm^2^), both with a *p* ≤ 0.05.

**Figure 5:**
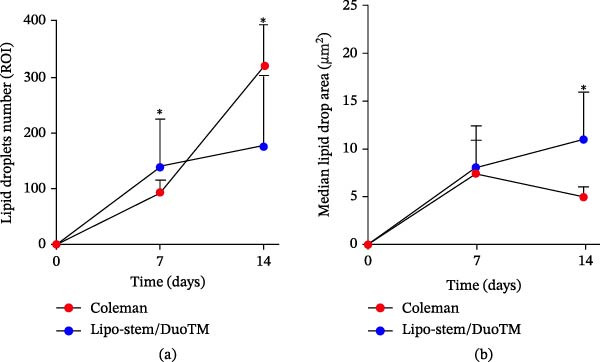
Lipid droplets’ number per ROI and mean lipid droplet area. (a) According to morphological evaluations, after 7 days of incubation, the number of lipid droplets per ROI was statistically significantly higher for the samples treated with Beauty and Lipo ‐Stem Duo than the Coleman samples. The situation reverses after 14 days of incubation; (b) According to morphological evaluations, after 14 days of incubation, the mean area of the lipid droplets is statistically significantly broader for the samples treated with Beauty and Lipo ‐Stem Duo than the Coleman samples. The increase in the droplet area dimensions is related to the reduction in the number of droplets and is compatible with adipocyte maturation.  ^∗^ *p* ≤ 0.05. (Beauty and Lipo‐Stem Duo systems, *N* = 3; Coleman’s processing, *N* = 3).

**Figure 6:**
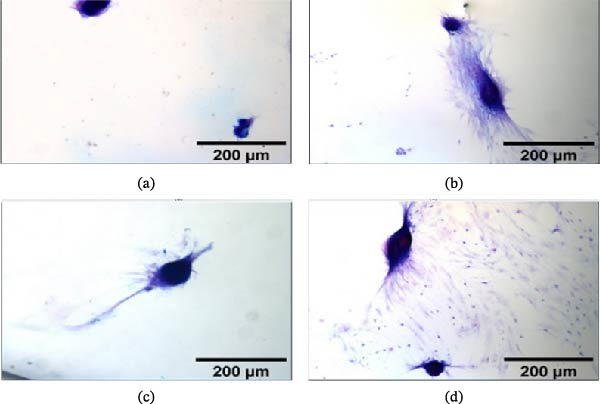
(a) Lipid droplets’ number per ROI and mean lipid droplet area. (b) According to morphological evaluations, after 7 days of incubation, the number of lipid droplets per ROI was statistically significantly higher for the samples treated with Beauty and Lipo ‐Stem Duo than the Coleman samples. The situation reverses after 14 days of incubation; (c) according to morphological evaluations, after 14 days of incubation, the mean area of the lipid droplets is statistically significantly broader for the samples treated with Beauty and Lipo‐Stem Duo than the Coleman samples. (d) The increase in the droplet area dimensions is related to the reduction in the number of droplets and is compatible with adipocyte maturation.  ^∗^ *p* ≤ 0.05. (Beauty and Lipo‐Stem Duo systems, *N* = 3; Coleman’s processing, *N* = 3).

As related to chondrogenic differentiation, at 14 days after incubation, the samples processed with Beauty and Lipo‐Stem Duo presented a larger amount of chondrocyte clusters (1,95 ± 0,18) than those of the samples processed with Coleman’s procedure (1,2 ± 0,4) with a *p* ≤ 0.05 (Figure 5). Moreover, the samples treated with Beauty and Lipo‐Stem Duo showed aggregates with a broader mean area than the aggregates obtained from CTRL samples. Still, the difference in size was not statistically significant (Figure 6).

**Figure 7:**
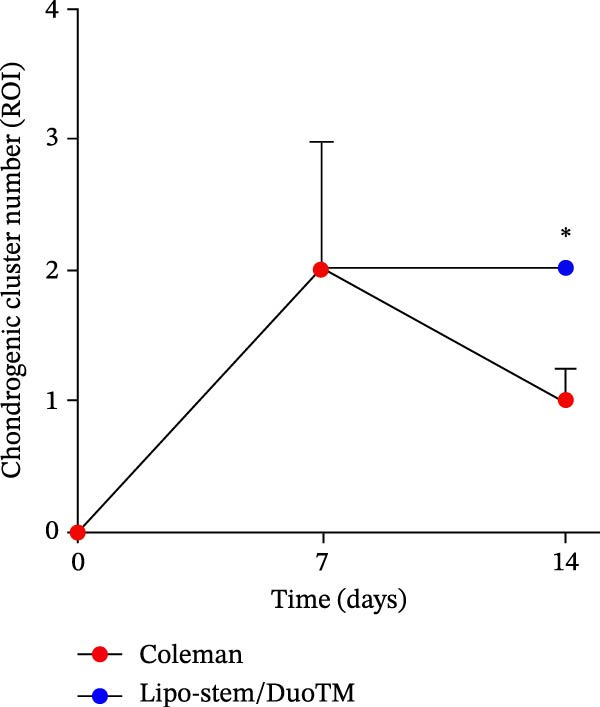
Chondrogenic clusters’ number per ROI. The number of chondrogenic clusters per ROI is statistically significantly higher for the samples treated with Beauty and Lipo‐Stem DuoTM concerning the Coleman samples after 14 days of incubation.  ^∗^ *p* ≤ 0.05 (Beauty and Lipo‐Stem DuoTM systems, *N* = 3; Coleman’s processing, *N* = 3).

**Figure 8:**
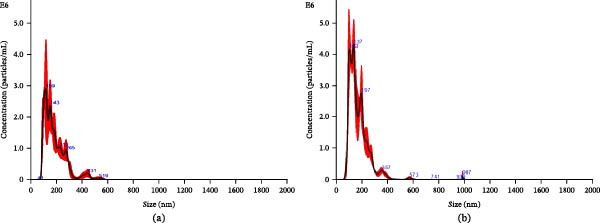
Nanoparticle tracking analysis (NTA) of EVs. (a) CTRL sample; (b) Sample treated with Beauty and Lipo‐Stem Duo. The dimensions of EVs are slightly lower in the samples treated with Beauty and Lipo‐Stem Duo than those processed using Coleman’s technique (Beauty and Lipo‐Stem Duo systems, *N* = 3; Coleman’s processing, *N* = 3).

Regarding osteogenic differentiation, both the CTRL samples and those treated with Beauty and Lipo‐Stem Duo presented calcium deposits (Figures 7 and 8). The dimensions of these areas were statistically significantly different after 7 days of incubation. In detail, after 7 days of incubation, the calcium deposit areas were broader for samples treated with Beauty and Lipo‐Stem Duo (15,965 µm^2^ ± 3101 µm^2^) than for CTRL samples (2328 µm^2^ ± 450 µm^2^) with a *p* ≤ 0.05.

### 3.5. EVs

Both the CTRL samples and the samples processed with Beauty and Lipo‐Stem Duo presented a high EV particle secretion. The mean EV size and the EV size mode (Table 1) did not differ among the samples. In the samples treated with Beauty and Lipo‐Stem Duo, the EV concentrations resulted in higher values than those in the CTRL samples (2,54 × 10^8^ for AWT vs. 1,72 × 10^8^ for CTRL).

**Table 1:** Measurement of EV size expressed as mean and mode of EV diameters (nm).

Group	Mean (±SEM)	Mode (±SEM)
Coleman	181.2 ± 15.6	132.5 ± 10.9
Beauty and Lipo‐Stem Duo	174.5 ± 6.0	133.3 ± 10.0

## 4. Discussion

The two new closed mechanical systems preserve adipocyte morphology and ADSC functional properties. The continuous washing step likely reduces biochemical residues and debris, potentially creating a more favorable environment for ADSC proliferation and differentiation.

Beauty and Lipo‐Stem Duo perform combined filtration and emulsification of harvested fat by continuous washing with a saline solution. Comparing Beauty and Lipo‐Stem Duo with other devices is not possible because of the absence of data in the literature and because the processing techniques implemented in other devices are different. Consistent with the statistical analysis, the growth advantage associated with continuous‑washing mechanical processing is limited to early time points (days 7 and 14). At day 21, the observed difference does not withstand multiple‑comparison correction and should be considered exploratory rather than confirmatory. It is important to note that several comparisons did not maintain statistical significance after applying multiple‑comparison corrections. Consequently, any observed differences should be regarded as indicative rather than conclusive.

Several studies emphasize the relevance of washing steps during fat processing. Solutions employed include saline [26], phosphate‐buffered saline [27], and glucose‐based formulations [28]. These steps aim to remove contaminants and improve graft quality [29].

Veronese et al. [30] reported device instructions relating to washing, and of the 12 instruments described, nine required at least a washing passage. Of these nine, two gave indications to use Ringer Lactate, three indicated saline solution, and for another two, the choice of the type of solution for the washing was left to the surgeons.

Research by Locke and de Chalain [31] underscores the necessity of washing to eliminate BWPs and residual drugs from harvested fat. Failure to remove these substances may provoke inflammatory reactions, characterized by vascular changes and immune cell infiltration. Such responses can compromise graft outcomes, including angiogenesis and tissue integration [32, 33].

It must also be stated that the formation of BWPs is a natural and obvious result of fat processing. The quantity of these BWPs is probably smaller than that resulting from fat harvesting. If washing is performed as the first step of fat processing, BWPs resulting from harvesting are removed. Still, the BWPs correlated with the other processing steps remain in the final product, which is reinjected into the body. Thus, it is fundamental to perform washing in the first phases of processing to remove the drug residues, which could alter the biochemical properties of the tissue. However, at least a second washing should be performed at the end of the processing to remove the BWPs that are related to the processing.

The continuous washing required by the Beauty and Lipo‐Stem Duo protocol solves this problem and determines a second, not secondary, effect. It guarantees soft processing, which results in a perfectly preserved morphology of the fat fragments, as highlighted in the experimental part of this study. It is well known that this does not imply the preservation of stemness properties nor the regenerative potential [18]. In this study, all the criteria for MSCs definition [24, 25] were verified, denoting how both processing systems do not alter the adipose tissue concentration of ADSCs and their properties. The initial cellular yield computation after ADSCs’ extraction confirmed the vitality of the ADSCs. The cellular yield obtained with the Beauty and Lipo‐Stem Duo systems was similar to that obtained with Coleman’s golden standard procedure. The subsequent cell growth analysis revealed a higher proliferation of the ADSCs extracted after the Beauty and Lipo‐Stem Duo processing, highlighting how these systems may offer some advantages compared with Coleman’s procedure.

It should be noted that the cellular growth ability after the new systems’ use resulted in greater than that observed after Coleman’s procedure, starting from the 7th day of culture. This suggests that the processing technique may influence growth. Thus, it is possible to hypothesize that the centrifugation of Coleman’s procedure, which even guarantees good vitality of ADSCs, may be associated with reduced ADSCs’ growth ability or that the new systems favor the growth ability of these cells. In any case, the result is potentially relevant to scientific research in the field of regenerative medicine.

The analysis of the differentiation experiments’ results offers further food for thought. In adipogenic differentiation, the broader lipid droplet dimensions of the cells processed with the Beauty and Lipo‐Stem Duo systems in all the experimental phases indicate a higher metabolic degree of these samples, with better and/or faster maturation of ADSCs in adipocytes, as per their natural behavior [34]. The increase in the lipid droplet number together with a temporary reduction in mean droplet size at 14 days is consistent with the early–intermediate phases of adipogenic differentiation. In this stage, ADSCs generate multiple small lipid droplets via de novo lipogenesis before they subsequently undergo fusion into larger unilocular structures. This may imply that fat grafting into an area where fat tissue is present could favor rapid integration and allow for effective neo‐adipogenesis.

In chondrogenic differentiation, the formation of rapidly growing chondrogenic depots and a chondrogenic matrix is of extraordinary interest in the field of orthopedics and, in general, in all regenerative medicine applications. After 14 days of incubation, the results were better than those of the samples treated with Coleman’s procedure. These results may suggest a faster and more effective integration action of the graft in the injection area.

The rapid differentiative response in the osteogenic differentiation experiments is of interest and warrants further investigation. The significant reduction in the calcium deposit areas must be investigated further. Nonetheless, the osteogenic differentiative capacity of the samples treated with the Beauty and Lipo‐Stem Duo systems is equitable to that of the samples processed with the golden standard Coleman system.

The analysis highlighted the statistically significantly higher growth ability of ADSCs in the samples treated with the Beauty and Lipo‐Stem Duo systems compared to Coleman’s samples. Moreover, the two systems permitted us to obtain samples presenting a higher concentration of EV than that of Coleman’s samples. These two data suggest that the cells obtained after the Beauty and Lipo‐Stem Duo processing may be more vital and active than the cells obtained from Coleman’s procedure.

It must be stated that the culture of ADSCs extracted from adipose tissue samples without a specific differentiative medium is known to result in cells with a fibroblast‐like shape, high proliferative potential, and multipotent capacity for lineage differentiation [4, 4, 35, 36]. This transition is also called dedifferentiation [37]. As the nature of induced differentiation depends on the specific cell culture model system employed, the in vivo differentiation is related to the reinjection site [5]. Poloni et al. [35] demonstrated that dedifferentiated cells of adipocytic lines could differentiate into adipogenic, osteogenic, chondrogenic, and neurogenic lineages. Consequently, their fields of application are vast, and their practical use, for instance, in tendon regeneration, is not to be excluded.

The obtained data suggest a robust healing phase after fat reinjection in the receiving site with the appropriate differentiative activity. In the present study, the fat was utilized for facial and neck lipofilling. For this reason, it was not possible to verify the healing times. However, in different applications, processed fat that is pure and active, such as that obtained with the Beauty and Lipo‐Stem Duo systems, might contribute to more rapid healing. Although our data demonstrate a significant improvement in adipogenic maturation, chondrogenic aggregate formation, and osteogenic potential with the new devices compared to the standard Coleman technique, we acknowledge that the specific molecular and cellular mechanisms mediating these effects remain incompletely understood.

Plausible hypotheses include the possibility that the gentler mechanical processing and continuous saline washing employed by the new systems may reduce cellular stress and the presence of inflammatory debris, thereby better preserving the viability and functionality of ADSCs and promoting a more favorable environment for cell proliferation and differentiation. Further studies focusing on molecular pathways and more in‐depth functional analyses are essential to elucidate the precise mechanisms involved.

In fat processing protocols, it is essential to report the RCF used during centrifugation. Merely indicating the rotations per minute (rpm) is insufficient for ensuring reproducibility and comparability across studies as RCF is directly dependent on the rotor radius. This lack of standardization renders interstudy comparisons potentially unreliable.

This issue is particularly relevant in light of findings by Prantl et al. [38], who demonstrated that even high centrifugal forces (up to 1600 RCF for 2 min) do not compromise cell viability nor violate the criteria for minimal manipulation of adipose tissue. Furthermore, it has been shown that sedimentation and mechanical processing methods significantly influence the composition of lipoaspirate and, consequently, the quality of the final product [39].

Finally, it is important to acknowledge certain limitations of the present study, particularly the assessment of adipocyte integrity based solely on histological morphological evaluations. While these analyses provide insights into the cellular structure, they do not offer direct information regarding cell functionality or viability. Recent studies have shown that mechanical processing, even under shear stress conditions, does not significantly alter the secretome of ADSCs, suggesting that cellular functionality may be preserved despite mechanical stress [40]. The absence of direct cell viability assays, such as live/dead staining or MTT analysis, represents another limitation of the present research. Although the morphological integrity of adipocytes, the cellular yield, the proliferative capacity of ADSCs, and their multilineage differentiation potential were thoroughly assessed, these parameters cannot fully replace quantitative viability testing. Therefore, our evaluation primarily relies on morphological and functional indicators rather than on direct metabolic or membrane‑integrity assays. Another limitation of this study is the small and nonuniform sample size (*n* = 17, of which only 5 were processed using the Coleman technique), which reduces the robustness of statistical comparisons, particularly when assessing subtle cellular differences in proliferation and differentiation potential. The small and unbalanced sample size (5 samples processed with Coleman’s technique vs. 12 samples processed with the new devices) represents a clear limitation of this study. As a consequence, the comparative analyses should be interpreted as preliminary in vitro evidence rather than definitive conclusions. Larger and more balanced cohorts will be necessary to confirm these findings and strengthen the statistical robustness of the comparative results. The mechanistic interpretation of our results was also reviewed to avoid overdoing it with speculative explanations. Although some biological patterns observed in the present study may suggest potential effects of gentler mechanical processing and continuous rinsing with saline on ADSC behavior, these considerations should be considered only as hypotheses based on the currently available literature and not as proven mechanisms.

The main limitation of this study is the absence of clinical feedback. Moreover, the analysis of more samples could strengthen the results obtained. Although the in vitro results are promising, the lack of clinical follow‐up regarding graft retention, tissue integration, or long‐term outcomes significantly limits the translational relevance of the present study. Further in vitro and in vivo studies are necessary to verify the potential translational relevance of the promising possibility that the results obtained have configured.

## 5. Conclusions

Data obtained in this work highlights that the Beauty and Lipo‐Stem Duo systems perfectly align with the new EU regulations and the minimal manipulation requisites, which maintain the morphology of the original tissue, its stemness properties, and its regenerative potential. The numerous possible application fields make them fascinating instruments for the esthetic and regenerative medicine.

## Author Contributions

Conceptualization: Sheila Veronese, Mario Goisis, Andrea Sbarbati, and Antonio Scarano. Methodology: Domenico Amuso, Riccardo Ossanna, Sara Ghazanfar Tehrani, Lorena Torroni, Patricija Kasilovska, Mario Goisis, Giamaica Conti, and Antonio Scarano. Validation: Riccardo Ossanna, Sara Ghazanfar Tehrani, Lorena Torroni, and Giamaica Conti. Formal analysis: Sheila Veronese, Riccardo Ossanna, Sara Ghazanfar Tehrani, Lorena Torroni, Giamaica Conti, and Antonio Scarano. Investigation: Riccardo Ossanna, Sara Ghazanfar Tehrani, Patricija Kasilovska, Mario Goisis, and Giamaica Conti. Data curation: Riccardo Ossanna and Sara Ghazanfar Tehrani. Writing – original draft preparation: Sheila Veronese. Writing – review and editing: Sheila Veronese, Riccardo Ossanna, Sara Ghazanfar Tehrani, Lorena Torroni, Patricija Kasilovska, Mario Goisis, Giamaica Conti, Andrea Sbarbati, and Antonio Scarano. Supervision: Andrea Sbarbati, and Antonio Scarano. Project administration: Andrea Sbarbati.

## Funding

The authors received no specific funding for this work. Open access publishing facilitated by Universita degli Studi Gabriele d’Annunzio Chieti Pescara, as part of the Wiley ‐ CRUI‐CARE agreement.

## Disclosure

All authors have read and agreed to the published version of the manuscript.

## Ethics Statement

Ethical review and approval were waived for this study because clinical practices were common practices at the De Clinic, not the object of the study. For this study, only the leftover biological material from surgery was analyzed. All the procedures were conducted in full compliance with the ethical norms and standards of the Helsinki Declaration of 1975, as revised in 1983.

## Consent

Written informed consent has been obtained from the patient(s) to publish this paper.

## Conflicts of Interest

The authors declare no conflicts of interest.

## Data Availability

The data will be made available upon request.
